# A Comparative Analysis of In Vivo-Generated and Artificial CoCrMo Wear Particles Created by High-Energy Ball Milling and the Buchhorn Method

**DOI:** 10.3390/ma18030643

**Published:** 2025-01-31

**Authors:** Adrian Buchholz, Rebecca Höpfer, Julia Becker, Vadym Voropai, Janett Schmelzer, Manja Krüger, Jessica Bertrand

**Affiliations:** 1 Experimental Orthopedics Research Unit, Orthopedic University Hospital Magdeburg, 39120 Magdeburg, Germany; 2 Institute of Materials, Technologies and Mechanics, Otto-von-Guericke-University Magdeburg, 39106 Magdeburg, Germany; 3 Center for Advanced Medical Engineering (CAME), Otto-von-Guericke University Magdeburg, 39106 Magdeburg, Germany

**Keywords:** CoCrMo, high-energy ball milling, Buchhorn method, particle morphology, biological testing

## Abstract

This study compares CoCrMo particles generated by a high-energy ball milling method with those produced by the established Buchhorn method and with in vivo-generated wear particles from periprosthetic tissue. CoCrMo particles were produced utilization both methods. The particle size distribution was analyzed using laser diffraction, and the morphology was examined through scanning electron microscopy (SEM). Energy-dispersive X-ray spectroscopy (EDS) provided a qualitative analysis of the material composition. The high-energy ball milling method yielded CoCrMo particles with a D50 of 7.92 µm, a significantly smaller value than the D50 of 88.30 µm observed for Buchhorn particles. The SEM analysis demonstrated that the particles produced by the ball mill have a roundish, red blood cell-like and irregular shape, similar to that observed in particles generated in vivo. In contrast, the particles produced by the Buchhorn method exhibited a flake-like and irregular morphology. The ball mill particles displayed a tendency towards agglomeration, which was also observed in particles generated in vivo. In conclusion, the ball mill method produces CoCrMo particles that closely resemble natural wear particles in terms of size and morphology. These particles provide a superior model for biological testing in comparison to those produced by the Buchhorn method. Furthermore, the ball mill method offers advantages in terms of reproducibility and scalability, making it a promising alternative for the generation of CoCrMo particles for orthopedic research.

## 1. Introduction

The use of cobalt–chromium–molybdenum (CoCrMo) alloys in arthroplasty is widespread, mainly due to their excellent mechanical strength, wear resistance, and corrosion resistance [1,2,3,4]. These properties make CoCrMo alloys a preferred choice for orthopedic implants, such as hip and knee replacements. However, there are concerns regarding the corrosion and the release of metal ions, as well as particles, into the surrounding tissue, which can lead to inflammatory reactions and adverse reactions [5,6,7,8,9,10,11,12,13,14]. The interaction between wear particles and the periprosthetic tissue is not fully understood yet, as it is difficult to mimic the in vivo process using in vitro techniques.

For instance, CoCrMo wear particles induce a strong inflammatory response by triggering macrophages to adopt a unique phenotype, termed corrosion-associated macrophages. The specific signaling pathways driving these changes remain not fully understood [15,16,17]. Additionally, the role of macrophages in modulating osteoclast activity and bone resorption near CoCrMo implants needs further study as the long-term effects are unknown [18]. Interestingly, the degree of toxicity of CoCrMo wear particles seems to vary significantly based on their physiochemical properties [19,20]. This emphasizes the need for particles that are as close to real wear particles as possible for the purpose of investigating them with the highest possible significance. Furthermore, the raising popularity of additively manufactured implants introduces new parameters like building orientation and microstructural differences, affecting the tribocorrosion properties and hence the properties of resulting wear. The resulting particles have shown different metal ion release profiles compared to wrought CoCrMo alloys [21,22,23].

This demonstrates that there is still a need for realistic CoCrMo abrasion particles and corresponding production methods in order to better investigate the open research questions identified. However, authentic in vivo wear particles are difficult to generate in vitro and their quality is hard to verify.

Authentic in vivo particles are usually obtained by means of chemical or enzymatic isolation and are adequately described in the scientific literature and in corresponding standards [24,25,26,27,28]. This is well suited for the analysis of morphology and size. In the context of biological experiments, however, purity and chemical integrity are essential to guarantee meaningful results, which is why these methods are not suitable for generating appropriate material.

Introduced in 1992, the Buchhorn method, named after the engineer who developed it, emerged as a pioneering technique in this realm, offering the most unparalleled approach to date for generating particles akin to natural wear particles from CoCrMo implants [29].

The Buchhorn method’s meticulous grinding process is based on the friction between small CoCrMo bars and rotating containers of the same material. It has been instrumental in numerous studies, primarily within the European research, where it is well-known due to its precision and reproducibility. The method has not only facilitated a deeper understanding of wear particle characteristics but has also contributed significantly to the field of orthopedic implant research and development [30,31,32,33,34,35,36,37,38,39].

However, this method was developed and used by a single working group, but never commercialized or replicated on a larger scale. And as the initial developers retired, the availability of these particles dwindled. Furthermore, today, 30 years after its development, advancements in technology have driven the exploration and testing of more efficient and precise methods for producing CoCrMo particles.

High-energy ball milling is a ball milling process in which a powder mixture placed in a ball mill is subjected to high-energy collisions with grinding balls [40,41]. For this study, Zirconia grinding balls were used, with the feed size of the powder reaching up to 5 mm in diameter. High-energy ball milling is commonly used to produce nanocrystalline metals or alloys in powder form [42,43,44]. This method’s ability to manipulate material properties, such as particle size, composition, and structure, makes it highly suitable for synthesizing metallic particles used in biomedical research. Additionally, this technique can be scaled and adapted to prepare powders of varied compositions, crucial for tailoring materials for specific experimental conditions [45,46,47]. For example, the microstructure and morphology of the resulting particles can be influenced by the size of the grinding balls [48] and the ball-to-powder ratio, respectively [49]. The working volume significantly influences the particles’ edges [50], whereas the milling time and its distribution is relevant for the resulting size distribution [50,51].

The objective of this study is to perform an analytical comparison of CoCrMo particles processed by the methods mentioned (high-energy ball milling and Buchhorn), using vivo-generated wear particles from the periprosthetic tissue as a reference. The aim is to compare their morphological properties to predict similar in vitro interactions with biological systems and therefore the theoretical suitability for further research into particle interaction with biological systems. We conclude this paper with a recommendation for future research meant to increase the comparability of the corresponding models and thus enable more significant results.

## 2. Materials and Methods

### 2.1. Materials

For the processing of particles, Co28Cr6Mo cast alloy according to the ISO Standard 5832-12 [52] was used. This alloy composition yields a beneficial combination of price and resulting properties, making it the most popular choice for metallic endoprosthetic implants [53,54].

#### 2.1.1. Buchhorn Method

This method, mentioned first in 1992 [29], describes the production of particles using multiple bars (h = 26.2 mm, Ø = 4.2 mm) and a cylindric container (h = 117 mm, Ø = 26.2/15 mm) of the same material. The bars are placed in the containers with ethanol, which are then sealed under pressure using silicone. The containers are subsequently rotated in a centrifuge machine for several days at room temperature, whereby the friction between the container and the bar generates fine wear particles. The containers are also rotated once a day by a few degrees around their longitudinal axis.

Since relevant parameters such as the rotation speed, rotation time, and centrifugal force are not known and do not emerge from Buchhorn’s publications, this method cannot be reproduced precisely.

#### 2.1.2. Ball Milling Method

**Figure 1:**
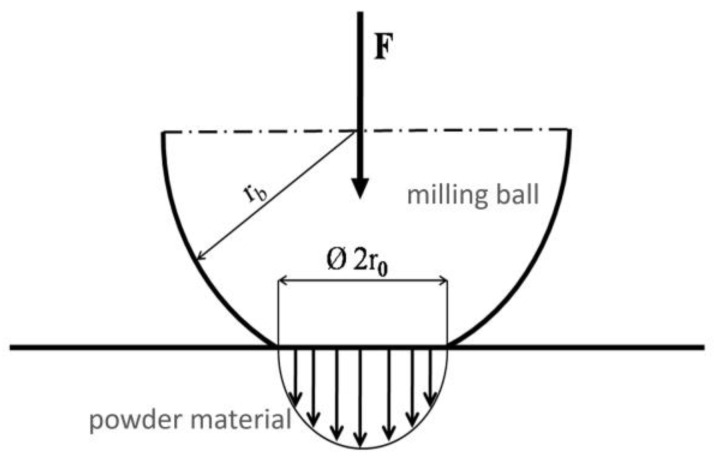
Mechanistic energy transfer from the milling balls to the powder material.

This method is based on a modern ball mill (High Energy Ball Mill E_MAX, Retsch, Haan, Germany) that enables a fast milling progress by using high-energy friction and impact forces. The working mechanical forces, which are transferred from the milling balls into the powder particles, are schematically drawn in Figure 1, while F represents the impact force, $r_{b}$ is the radius of the milling ball, and 2$r_{0}$ is the mechanical force affecting the power by the Hertzian pressure. A Zirconia grinding bowl with a volume of 50 mL was filled with 30 g of CoCrMo powder with a particle diameter of 20 to 40 µm and 110 g of Zirconia grinding balls with a diameter of 0.5 mm, and was topped with 15 mL of isopropanol. The grinding bowl then was rotated at a speed of 2000 rpm. In contrast to the Buchhorn method, the alignment of the container does not rotate, which additionally increases the friction between the container and its contents, thus increasing the induced grinding energy and making the grinding process more efficient. After a grinding time of 240 min, the contents were removed, separated from the grinding balls by sieving, and dried in a desiccator. These milling parameters were derived from former experimental tests and empirical values.

### 2.2. Experimental Methods

#### 2.2.1. Determining the Size Distribution Using Laser Diffraction

The size distribution was determined using a Mastersizer 3000E (Malvern Panalytical, Malvern, UK), which is based on the principles of laser diffraction. The particle samples were dispersed in distilled water and penetrated by two lasers with wavelengths of 470 nm and 632.8 nm, respectively. Forward scattering, side scattering, and backscattering were detected and examined according to Mie and Fraunhofer.

Twenty measurements with a duration of 10 s each were taken per sample and the mean value of these measurements was used for the evaluation.

Particles from the periprosthetic tissue were not analyzed by this method because it was not possible to completely isolate them from the tissue. Instead, a qualitative size comparison was made based on the SEM images.

#### 2.2.2. SEM and EDS Examination

Scanning Electron Microscopy (SEM) examinations in this study were performed with a FEI XL30 ESEM-FEG (FEI/Philips, Hillsboro, OR, USA) equipped with a secondary electron (SE) and backscattered electron (BSE) detector operating at 10 kV in an environmental pressure mode of ~$5\times 10^{-5}$ mbar. The chemical composition of the CoCrMo particles was verified using energy-dispersive X-ray spectroscopy (EDS, EDAX-AMETEK GmbH, Weiterstadt, Germany) at an accelerating voltage of 10 kV. The particle samples were spread out on high-purity, double-sided, conductive carbon adhesive pads, sputtered with gold and examined regarding size, morphology, and chemical composition.

During revision procedures, small samples of periprosthetic tissue were obtained in some patients and underwent routine processing for paraffin block preparation. For this study, microscopic sections of 4 µm thickness were sputter-coated with gold and examined for presence of CoCrMo wear particles.

The relatively low accelerating voltage of 10 kV was used for EDS as the small particle diameter would likely be penetrated by electrons with higher energies [55].

### 2.3. Statistics

The statistical analysis and the graphical representation of results was performed using GraphPad Prism V.9 (GraphPad Prism Software, La Jolla, CA, USA). If not stated otherwise, the descriptive data are mean ± standard error of mean (SEM); the level of significance was set at *p* < 0.05 for all statistical tests. The normality of data distribution was verified using Shapiro—Wilk test; two-way ANOVA with Šídák’s multiple comparisons correction was used for comparing data from different groups. 

## 3. Results and Discussion

### 3.1. Laser Diffraction

**Figure 2:**
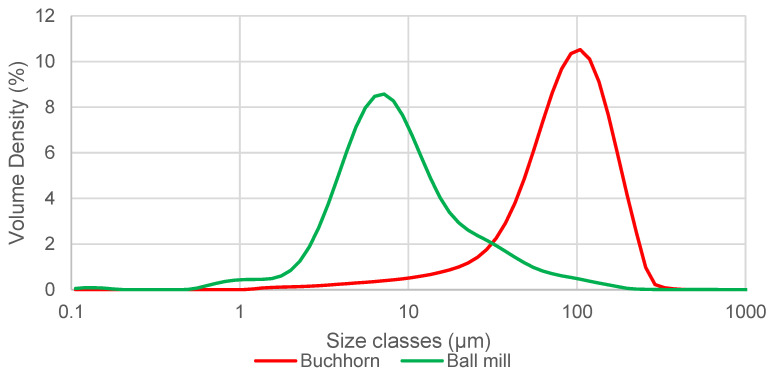
Size distribution of particles processed using the high-energy ball mill and Buchhorn method, respectively.

Figure 2 shows the volume distribution of particles generated with the high-energy ball mill (green) and the Buchhorn method (red), respectively. Whereas the amplitude is merely a function of the particle concentration in the dispersant, the shift on the *x*-axis indicates a significant difference regarding the particle size distribution between both methods. The peak for the size of the ball mill particles is at 7 µm, whereas the peak size of the Buchhorn particles lies at 100 µm. This observation is in line with the size and size range obtained using SEM (compare “D50” and “size range” in Table 1).

**Figure 3:**
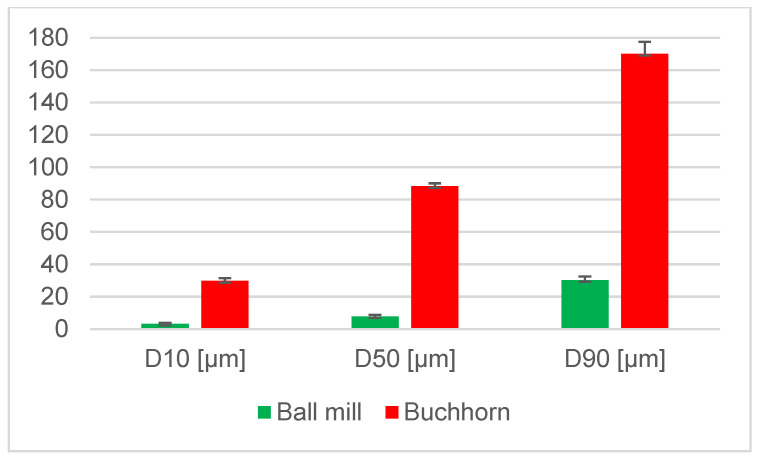
Measurement results from the particles generated using the high-energy ball mill and Buchhorn method, respectively, obtained using laser diffraction examination. All values in µm; *n* = 10 per graph.

Figure 3 shows the size of the particles produced using the high-energy ball mill compared to the Buchhorn method. The resulting size of the Buchhorn particles (D50 = 88.30 µm) is approximately one order of magnitude larger than that of the ball mill particles (D50 = 7.92 µm). This difference is significant (*p* < 0.0001).

The literature reports that CoCrMo wear particles from MoM (metal-on-metal) bearings generated in simulator tests account for a maximum dimension of approximately 46 to 52 nm, with round to oval shapes and some needle-shaped particles depending on the wear cycle [56]. Others found particles from MoM hip simulator tests to have an average length from 15 to 80 nm [57] and a maximum length 40 to 120 nm, respectively [58]. Doorn et al. isolated particles from the periprosthetic tissue of MoM THR revision patients; most particles had a maximum dimension smaller than 50 nm with the overall range spanning from 6 to 834 nm [25]. These observations place the majority of particles from MoM bearings within a range of 40–80 nm, which is approximately within the same range as the ball mill-generated particles. Publications about CoCrMo particles from metal-on-polyethylene (MoPE) bearings specifically are scarce. This might be related to their low availability and prevalence, which results from the tribological conditions of a hard–soft pairing, as is a given with MoPE. Milošev et al. [59] analyzed the periprosthetic tissue from MoM and MoPE implants and found nanosized CoCrMo particles in the MoM cohort and no metallic particles in the MoPE cohort, aligning with the thesis regarding minimal abrasion at the harder partner in hard–soft bearings. As particle size is one of the most influential parameters regarding its interaction with tissue and cells [60,61], it is legitimate to assume that the interaction of the ball mill particles with tissue is closer to the in vivo situation than the Buchhorn particles due to the higher proximity regarding the average particle size.

**Figure 4:**
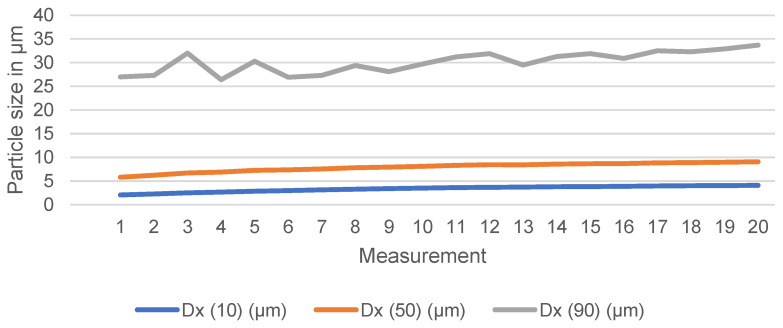
Measurement results of the same ball mill sample over 20 measurements of 10 s each with regard to the quantitative output parameters D10, D50, and D90 in µm, plotted as approximated curve.

A tendency towards agglomeration was observed for particles generated using the ball mill method. This is shown in Figure 4, which plots the measurements of the very same sample over 20 measurements of 10 s each, exhibiting an upward trend, an evident indication of the formation of agglomerates. For this reason, the “size range” was introduced as an additional control parameter to verify the laser diffraction results. This is based on manual measurements of the particles visible in the SEM image and is described in 3.2. The Buchhorn particles did not exhibit analogous agglomeration behavior.

**Figure 5:**
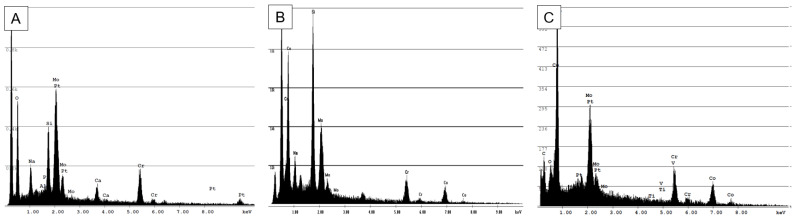
Representative EDS spectra; particle source: (**A**) in vivo, (**B**) high-energy ball mill, (**C**) Buchhorn method; acceleration voltage: 10 kV.

**Figure 6:**
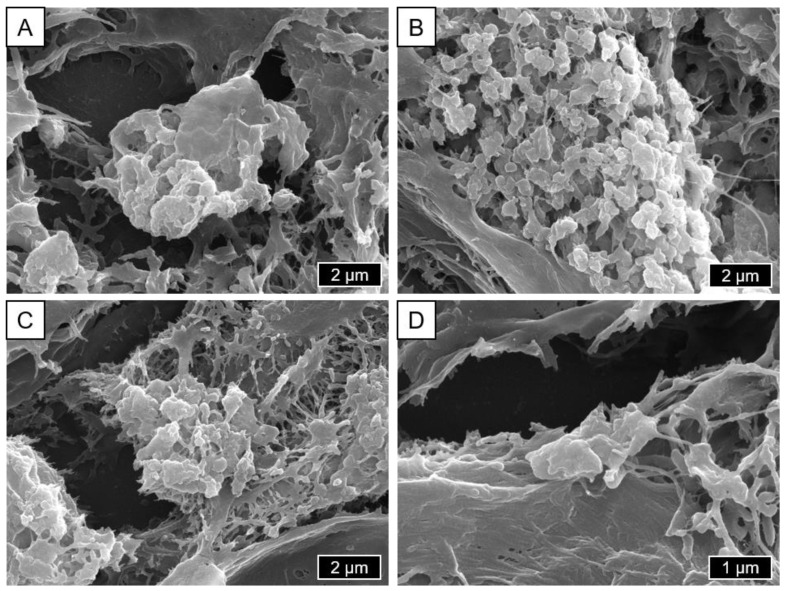
Representative SEM/SE images showing pathological particles from the periprosthetic tissue obtained during a hip revision surgery (**A**–**C**: 10,000×; **D**: 20,000×).

The bias of the ball mill particles towards agglomeration, which is quantitatively displayed in Figure 4 and qualitatively shown in Figure 6, is also found in the in vivo-generated particles (Figure 5) and is therefore likely a regular phenomenon. Such agglomeration can be attributed mainly to attractive surface forces (electrostatic charges) that gradually overpower repulsive interactions and van der Waals forces acting over short distances and favoring particle adhesion. The surface properties of particles have a direct influence on their agglomeration state and vice versa [62]. In this case, the tendency to agglomerate could be linked to an increased surface charge, a broader size distribution and, derived from that, a different interaction with their environment [63]. The agglomeration of the ball mill and in vivo-generated particles could therefore be interpreted as an indication of similar properties in terms of surface charge and surface parameters or as another distinctive parameter when comparing these particles to the ones generated using the Buchhorn method. However, further investigations would be necessary to determine the exact cause.

### 3.2. SEM and EDS

SEM and EDS analysis were conducted on artificial particles generated using the high-energy ball mill and the Buchhorn method. For reference, particles from periprosthetic tissue were also analyzed regarding size range and geometry. The shape and morphology of particles are further distinctive parameters for the interaction with cells, especially the phagocytosis of the particles. The description of the topography is based on the recommendations of the ASTM standard F1877-16 [64].

All the particles shown consist of CoCrMo alloy, according to the corresponding EDS spectra (Figure 5). The low cobalt content of the abrasion particles compared to the initial content is a well-known phenomenon [19,56,65]. It can be attributed to two causes. On the one hand, the outermost oxide layer, which decomposes first during abrasion, consists mainly of chromium oxide, as chromium is a preferred oxide former [66,67]. This is particularly relevant in the case of intermittent loading, since the surfaces have the necessary time to repassivate. As the artificial particles were manufactured continuously, the effect could be less pronounced with them. Furthermore, cobalt has a preferential tendency to be dissolved as an ion in physiological solutions [19,68]. Since the investigated particles were not stored in a corresponding solution, this effect cannot be observed, but this is to be expected.

Figure 6 shows representative images of the in vivo-generated particles. These are predominantly round with an agglomerated red blood cell-like form and an even shape distribution. The particles are not uniformly distributed in the tissue, but are found in agglomerations. The surface is smooth, with the corners rather soft or obtuse-angled. The size range of the visible particles ranges from 0.1 to 5 µm.

This is in line with former work. Catelas et al. described CoCrMo wear particles, especially after shorter implantation periods, as nanometer-sized, with a round to oval form [69]. Milošev and Remškar reported similar results, describing globular particles up to 90 nm in diameter and smaller, needle-shaped particles (40–120 nm) [58]. Topolovec et al. analyzed tissue samples from 31 patients and found a similar formation of clusters without specifically describing their form [70]. On this basis, the SEM images displayed (Figure 6) show abrasion particles from the earlier abrasion phase and can therefore be regarded as representative.

**Figure 7:**
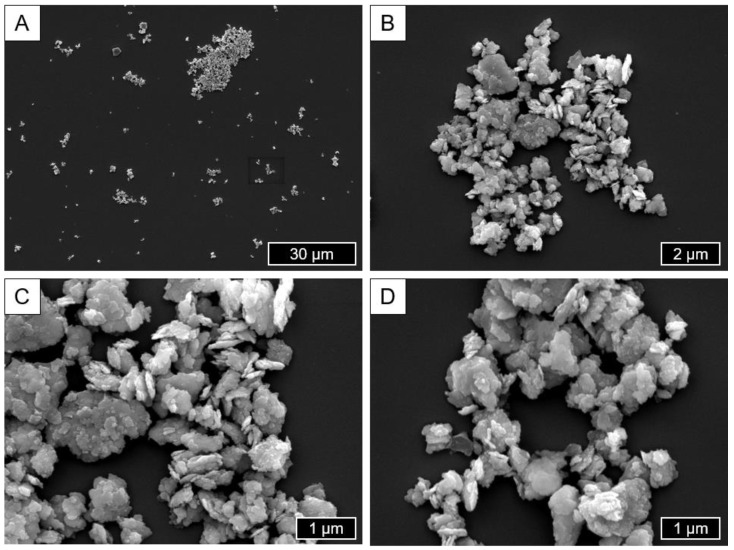
Representative SEM/SE images showing particles generated using the high-energy ball mill (**A**: 1000×; **B**: 10,000×; **C**,**D**: 20,000×).

Figure 7 shows representative images of particles generated using the method based on a high-energy ball mill described in Section 2.1.2. These predominantly exhibit a roundish shape distribution with an agglomerated red blood cell-like form. The size distribution is homogeneous. The particles form agglomerates, but this might be related to the preparation method including the air-drying of small amounts of the particle-containing solvent and may therefore not be representative due to the capillary effect. The surface is smooth, and the corners rather soft or obtuse-angled. The size range of the visible particles ranges from 0.5 to 5 µm.

**Figure 8:**
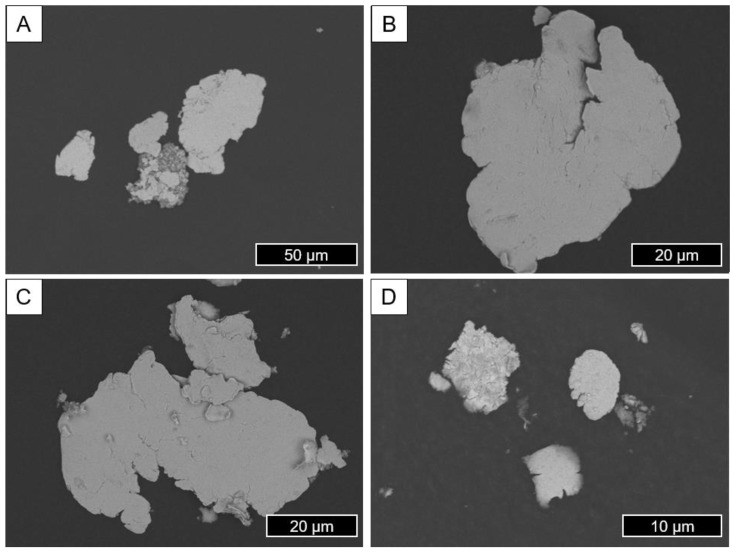
Representative SEM/BSE images showing particles made using the method according to Buchhorn et al. [13] (**A**: 650×; **B**,**C**: 1500×; **D**: 3500×).

Figure 8 shows representative images of particles generated using the method based on Buchhorn et al. [29] described in Section 2.1.1. These are formed like a smooth flake and have an irregular shape distribution; the thickness especially is relatively small, accounting for a disk-like geometry. There seems to be no tendency towards agglomeration, which is particularly interesting in a direct comparison to the ball-milling particles which were similarly prepared for the SEM evaluation. This confirms the assumption already made in Section 3.1 regarding different agglomeration tendencies. The surface is smooth, and the corners are rather fissured and partially torn. The size range of the visible particles ranges from 5 to 60 µm.

Figure S1 within the Supplementary Materials shows additional height profiles of both artificially generated cohorts, underlining the findings from the SEM images by introducing another perspective. More specifically, the flake-like shape and the flatness of the Buchhorn particles, especially when compared to the spherical and agglomerating high-energy ball mill particles, becomes evident.

Mathaes et al. observed a reduced particle intake of macrophages for elongated, non-spherical particles when compared to spherical particles [71]. Yang et al. analyzed the influence of the form of hydroxyapatite particles on the uptake by bone mesenchymal stem cells and proved that spherical particles are absorbed more efficiently compared to rod-shaped ones [72]. This shows that the interaction of cells and particles depends on the morphology of the latter. Since the high-energy ball mill-generated particles share more similarities in morphology with the in vivo-generated particles than the Buchhorn particles, it can be assumed that their biological behavior is more similar and comparable to the same extent.

The particle size is another distinctive factor affecting the particle–cell interaction. The particle uptake tends to be qualitatively and quantitatively inverse to the particle size whereby even small differences can cause significant effects and the specific cell type has a big impact on the individual mechanism [73,74,75,76]. Thus, a different particle uptake of physiological and high-energy ball mill particles is conceivable. However, due to the significantly smaller size deviation in comparison to the relationship between physiological and Buchhorn particles, this difference can definitely be considered to be just as significantly small. Consequently, a more realistic behavior of the high-energy ball mill particles in biological tests can be expected. Nevertheless, further research is necessary to enable a conclusive assessment.

The surface quality of the examined particles is evaluated as a further comparison parameter. The smoothness of the particle surface has an influence on protein binding and thus on cell interaction; in general, particles with a smoother surface are taken up more efficiently by cells due to a higher protein adsorption [77]. All three cohorts exhibit smooth surfaces according to the SEM images. Consequently, no differences are to be expected on the basis of this characteristic. The ball mill particles are partially covered with nanosized particles of the same material that have small geometries and are connected to the surface (Figure 7C,D), a feature that was not found neither in in vivo-generated nor Buchhorn particles.

Table 1 represents an overview of the results obtained using laser diffraction and SEM on the pathological and artificial particle cohorts.

Lastly, it should be mentioned that the actual model suitability of the particles strongly depends on the individual experiment design and can therefore only conclusively be assessed in each experiment with relevant cell line and setting. Different cell lines might respond differently to varying particle sizes and shapes and therefore must be assessed individually. The subjectivity of microscopic examination with regard to the selection of suitable ROIs is also an inherent limitation, as it cannot be precluded that all relevant properties were detected.

## 4. Conclusions

The present study evaluated a method of producing artificial CoCrMo wear particles for biological research using a high-energy ball mill. Our findings indicate that this method is able to produce particles with a different size, size distribution (D50 of 7.92 µm vs. 88.30 µm) and morphology (red blood cell-like vs. flake-like), which closely resemble in vivo-generated particles found in periprosthetic tissues. In terms of reproducibility and availability, the ball mill method presents an efficient and scalable approach for producing CoCrMo particles, as all of the parameters used are available for cell–particle interaction studies.

Future research should focus on further refining this technique and exploring its application in biological in vitro and in vivo studies to fully harness its potential for orthopedic implant research.

## Figures and Tables

**Table 1:** An overview of qualitative properties of the examined particle cohorts obtained using SEM and laser diffraction examination. D50 could not be determined for the in vivo-generated particles due to imperfect isolation methods.

	In Vivo-Generated	Ball Mill	Buchhorn
**Shape**	Spherical, agglomerated red blood cell-like form	Spherical, agglomerated red blood cell-like form	Flake-like
**(distribution)**	Irregular	Irregular	Irregular
**Corners**	Round	Round	Round, fissured
**Surface**	Smooth	Smooth	Smooth
**D50 in µm**	-	7.92	88.30
**Size range in µm**	0.1 to 5	0.5 to 5	5 to 60

## Data Availability

The data presented in this study are available on request from the corresponding author.

## Supplementary Materials

The following supporting information can be downloaded at https://www.mdpi.com/article/10.3390/ma18030643/s1: Figure S1: Representative images and height profiles of representative particles from the artificially generated particle cohorts; top: Buchhorn method, bottom: high-energy ball mill.
