# Supplementary figures and images for: A Comparative Analysis of In Vivo-Generated and Artificial CoCrMo Wear Particles Created by High-Energy Ball Milling and the Buchhorn Method
Source: Materials (Basel). 2025 Jan 31;18(3):643. doi: 10.3390/ma18030643 (PMC11819799; doi:10.3390/ma18030643)

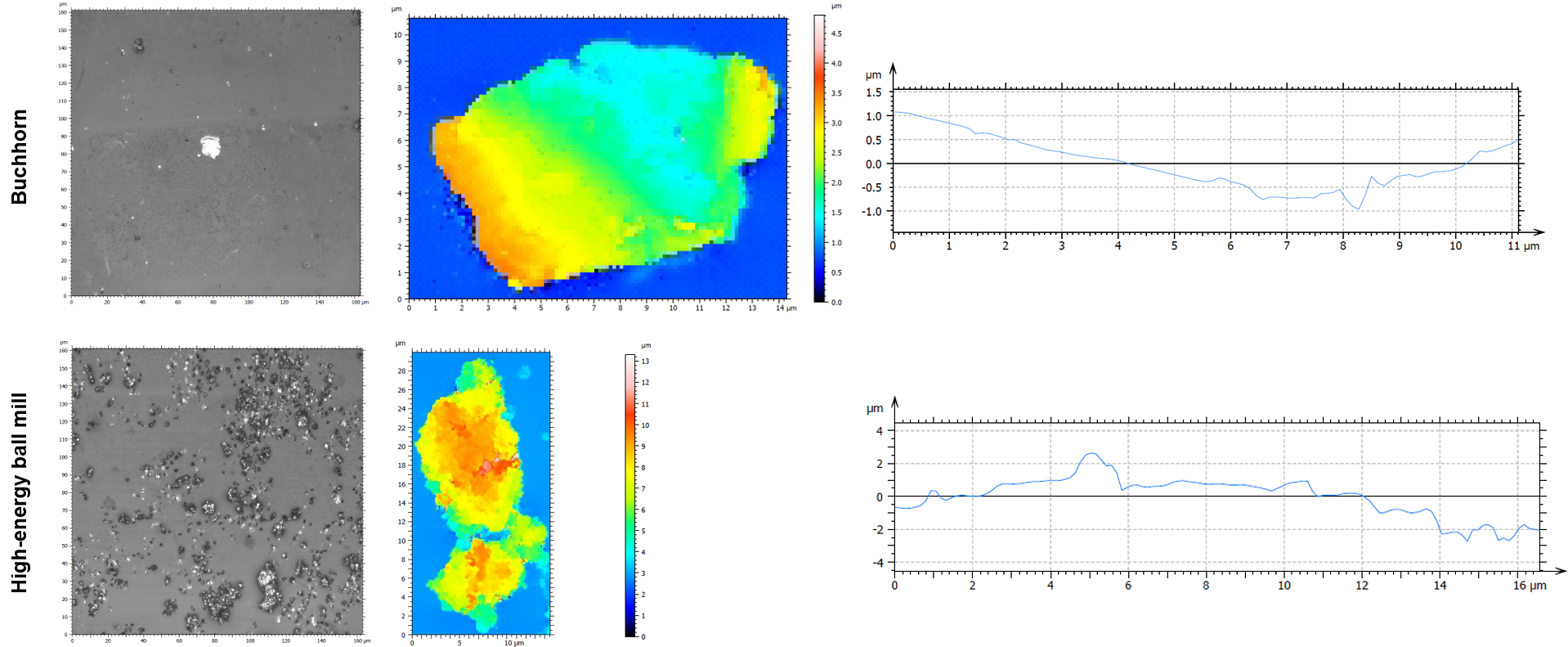

Supplement: Supplementary file 1 [file materials-18-00643-s001.zip › materials-3404780-supplementary.png]
